# Women with knee osteoarthritis have more pain and poorer function than men, but similar physical activity prior to total knee replacement

**DOI:** 10.1186/2042-6410-2-12

**Published:** 2011-11-10

**Authors:** Shalome M Tonelli, Barbara A Rakel, Nicholas A Cooper, Whitney L Angstom, Kathleen A Sluka

**Affiliations:** 1 College of Nursing, University of Iowa, Iowa City, IA, USA; 2 Physical Therapy and Rehabilitation Science Graduate Program, University of Iowa, Iowa City, IA, USA

## Abstract

**Background:**

Osteoarthritis of the knee is a major clinical problem affecting a greater proportion of women than men. Women generally report higher pain intensity at rest and greater perceived functional deficits than men. Women also perform worse than men on function measures such as the 6-minute walk and timed up and go tests. Differences in pain sensitivity, pain during function, psychosocial variables, and physical activity levels are unclear. Further the ability of various biopsychosocial variables to explain physical activity, function and pain is unknown.

**Methods:**

This study examined differences in pain, pain sensitivity, function, psychosocial variables, and physical activity between women and men with knee osteoarthritis (N = 208) immediately prior to total knee arthroplasty. We assessed: (1) pain using self-report measures and a numerical rating scale at rest and during functional tasks, (2) pain sensitivity using quantitative sensory measures, (3) function with self-report measures and specific function tasks (timed walk, maximal active flexion and extension), (4) psychosocial measures (depression, anxiety, catastrophizing, and social support), and (5) physical activity using accelerometry. The ability of these mixed variables to explain physical activity, function and pain was assessed using regression analysis.

**Results:**

Our findings showed significant differences on pain intensity, pain sensitivity, and function tasks, but not on psychosocial measures or physical activity. Women had significantly worse pain and more impaired function than men. Their levels of depression, anxiety, pain catastrophizing, social support, and physical activity, however, did not differ significantly. Factors explaining differences in (1) pain during movement (during gait speed test) were pain at rest, knee extension, state anxiety, and pressure pain threshold; (2) function (gait speed test) were sex, age, knee extension, knee flexion opioid medications, pain duration, pain catastrophizing, body mass index (BMI), and heat pain threshold; and (3) physical activity (average metabolic equivalent tasks (METS)/day) were BMI, age, Short-Form 36 (SF-36) Physical Function, Kellgren-Lawrence osteoarthritis grade, depression, and Knee Injury and Osteoarthritis Outcome Score (KOOS) pain subscale.

**Conclusions:**

Women continue to be as physically active as men prior to total knee replacement even though they have significantly more pain, greater pain sensitivity, poorer perceived function, and more impairment on specific functional tasks.

## Background

Osteoarthritis (OA) of the knee affects a greater percentage of women than men and can severely impact a person's function and quality of life [1,2]. When osteoarthritis becomes severe, total knee replacement (TKR) is indicated to improve pain and function of the affected joint. Prior to TKR, women have greater pain than men when measured using self-report surveys such as the Western Ontario and McMaster Universities Arthritis Index (WOMAC) and Knee Society Scale [3-6]. A greater decrease in perceived function is also reported for women when compared with men with knee osteoarthritis as measured by the Knee Society Score and the WOMAC [3-6]. Similarly, physical function tests, such as the 6-minute walk test and the timed up and go, show worse scores for women with knee osteoarthritis when compared to men [7,8]. This decrease in function in women is associated with lower quadriceps strength as measured by isometric maximal voluntary contraction [8,9].

It is known that healthy women and men differ on quantitative sensory testing measures including pressure pain threshold, heat, and cold measures [10-13]. It is unknown if those differences in pain sensitivity persist in populations with chronic knee pain. Women and men may also differ on psychosocial factors. Women have higher rates of depression whether they have chronic pain or are pain free [14]. While pain catastrophizing is predictive of future chronic pain development, disability and pain intensity [15-17], gender differences in pain catastrophizing in people with late stage osteoarthritis are not known. One possibility is that increased pain and reduced function in women can be attributed to differences in psychosocial variables.

It would be expected that since perceived function and physical function tests are reduced in women with osteoarthritis [3,5-8] that daily physical activity measured by accelerometry would be similarly decreased. Accelerometry in people with early osteoarthritis show less time spent doing vigorous activities with men spending more time doing moderately and vigorously intense activity than women [18,19]. Accelerometry in people with late osteoarthritis show also reductions in physical activity; these reductions also occur at lower activity levels [20]. However, it is not clear if these sex differences in physical activity also occur when osteoarthritis becomes more severe prior to surgery for total knee replacement, and if pain during function or if psychosocial variables contribute to physical activity levels.

The purpose of the current study was to determine if (1) women and men with late stage OA differ significantly on pain at rest and during movement, pain sensitivity using quantitative sensory testing, function, psychosocial variables, and physical activity levels immediately prior to TKR, and (2) which variables explain the differences in pain, function and physical activity.

## Methods

Subjects were recruited from a large teaching hospital through the orthopedics joint replacement clinic and were invited to participate if they were indicated for unilateral TKR for osteoarthritis. Data collection occurred from June 2008 through to December 2010. Eligible subjects were approached by a study recruiter and the informed consent process was completed. Consenting subjects were screened for sensation and ability to follow directions using three items from the Mini Mental State Exam (MMSE). A total of 385 subjects were approached and 208 participated in the study (138 women and 70 men). A total of 96 declined to participate and 81 did not meet eligibility criteria due to: other severe untreated painful conditions (N = 26), stroke or central nervous system lesion (N = 17), scheduling issues (N = 17), sensory impairment (N = 10), current prisoner (N = 7), cognitive impairment (N = 2), or wheelchair bound (N = 2). Refusal rates were not significantly different (*P *= 0.10) between women (22.7%) and men (30.7%). Eligible and consenting subjects completed the research testing during their preoperative investigation clinic visit, which typically occurred 1 week prior to the surgery date.

The outcome measures were collected during the visit by a trained research assistant who was a registered nurse or physical therapist. We assessed (1) pain using self-report measures (Brief Pain Inventory (BPI), Knee Injury and Osteoarthritis Outcome Score (KOOS), Short-Form 36 (SF-36)) and with a 0 to 20 numerical rating scale (NRS) at rest and during function tests, (2) pain sensitivity using quantitative sensory measures, including pressure pain thresholds, heat pain thresholds, and heat tolerance, (3) psychosocial variables including depression (Geriatric Depression Scale), anxiety (State Trait Anxiety Inventory), pain catastrophizing (Pain Catastrophizing Scale), and social support (Social Provisions Scale), (4) function with self-report measures (KOOS, SF-36), specific function tasks (timed walk, maximal active flexion and extension), and (5) physical activity using accelerometer (average metabolic equivalent tasks (METS)/day and average steps/day).

### Outcome measures

#### Demographics

The following information was collected from subjects and their medical records: gender, age, race, marital status, education, income, duration of knee pain, OA grade (Kellgren-Lawrence), pain or OA in the contralateral knee, height and weight, and analgesia intake.

### Pain

#### 0 to 20 NRS

Pain intensity at rest and during flexion, extension, and walking was measured on a 0 to 20 point NRS with 0 anchored with 'no pain' and 20 anchored with 'most intense pain imaginable'. NRS is strongly correlated with other pain scales such as visual analog scales (r = 0.91 to 0.95) [21-23] and is associated with higher compliance and lower failure rates in older adults [22].

#### BPI

The BPI was originally designed to measure pain in cancer patients, but has been determined to be a valid tool for pain measurement in other types of chronic pain including musculoskeletal pain in older adults [24]. The BPI intensity scale consists of four items where subjects rate their pain intensity (0 = no pain, 10 = pain as bad as you can imagine) while the BPI interference scale has seven items asking subjects to rate pain interference in aspects of daily functioning (0 = does not interfere, 10 = interferes completely). The BPI has adequate internal consistency for both the intensity score (0.85) and the interference score (0.88) [24] as well as acceptable test-retest reliability (r = 0.58 to 0.95) and validity (Cronbach α ≥ 0.85) [25].

### Hyperalgesia (quantitative sensory testing)

#### Pressure pain threshold (PPT)

Pressure was applied to sites around the operative knee with an electronic pressure algometer (Somedic, Somedic AB, Box 194, SE-242 22 Hörby, Sweden). Pressure was applied using a 1 cm^2 ^surface at a rate of 40 kPa/s. The subject was instructed to push a button when the pressure sensation first became painful.

#### Heat pain threshold (HPThr)

Contact heat was applied to sites around the operative knee with a 16 mm × 16 mm surface thermode (Medoc TSA; Medoc Ltd 1 Ha'dekel St., Ramat Yishai 30095, Israel) that increases in temperature at a rate of 1°C/s. The subject was instructed to click a button when the heat sensation first becomes painful (heat pain threshold).

#### Heat pain tolerance (HPTol)

After the heat threshold test, the temperature returned to baseline. The thermode device again provided increasing heat and subjects were instructed to click the button when the heat reached the most heat tolerable. The device safety mechanism is programmed to stop prior to skin damage.

Inter-rater reliability was determined at the beginning of the study and as needed throughout the study for pain sensitivity measures. Intraclass correlations ranged from 0.87 to 0.97 for pressure pain threshold, 0.70 to 0.92 for heat pain threshold, 0.72 to 0.98 for heat pain tolerance.

### Perceived pain and function

#### KOOS

The KOOS was developed from the WOMAC as a knee-specific self-report assessment instrument and has been validated in subjects with knee OA [26]. The KOOS consists of five subscales: (1) Pain, (2) Other symptoms, (3) activity in daily living (ADL), (4) function in sport and recreation (Sport/Rec), and (5) knee related quality of life (QoL). The last week is taken into consideration when answering the questions. Standardized answer options are given (five Likert boxes) and each question receives a score from 0 to 4. The scores are transformed to a 0 to 100 score (0 = extreme symptoms to 100 = no symptoms) for each subscale.

#### SF-36 Health Survey

The SF-36 contains 36 questions to measure self-reported functional health and well-being. It is a practical, reliable (α > 0.85), and valid measure of physical and mental health [27]. The SF-36 provides scores for each of eight health domains: (1) Physical Function, (2) Role - Physical (limitations due to physical health status), (3) Bodily Pain, (4) General Health, (5) Vitality, (6) Social Functioning, (7) Role - Emotional (limitations due to mental health status), and (8) Mental Health. Items are transformed to a 0 to 100 (0 = worse health to 100 = perfect health) score.

### Function tests

#### Range-of-motion measurements

Maximum active flexion and extension were measured using a long-arm goniometer. The subject was placed in a supine position on an examination table. The goniometer was aligned with the stationary arm along the lateral midline of the femur toward the greater trochanter, the axis at the lateral epicondyle of the femur, and the moving arm along the lateral midline of the fibula aligned with the fibular head and lateral malleolus. For active extension a towel roll was placed under the ankle to allow for the greatest extension. Goniometer measures have concordant validity with radiography of 0.97 to 0.99 [28-31]. Intraclass correlation coefficients (ICCs) were 0.52 to 0.69 for active extension and 0.91 to 0.97 for active flexion. The lower ICC scores for active extension are related to the fact that extension scores range from 0 to 3 degrees in healthy individuals and are consistent with studies testing reliability of extension and flexion using a long arm goniometer [32].

#### Gait speed test

Subjects were asked to walk 'as fast as you safely can' for 15 s down a straight hallway with the research assistant timing them with a stopwatch and measuring the distance traveled in inches. ICC scores ranged from 0.88 to 0.99 for gait speed distance. Gait speed has an inter-rater agreement of 89% to 94% [33] and moderate test-retest reliability (ICC = 0.56) [34].

### Psychosocial variables

#### Geriatric Depression Scale - Short Form (GDS-SF)

The GDS-SF is a depression screening tool that has five self-report items with a response format of 'yes' or 'no'. The five-item GDS-SF has been validated in many different older populations and has as sensitivity of 0.94, specificity of 0.81, and good test-retest reliability (κ = 0.84) [35,36]. Scores of ≥ 3 were classified as positive for depressive symptoms.

#### State Trait Anxiety Inventory (STAI)

The STAI is a self-report tool that includes separate measures for state and trait anxiety [37]. State anxiety reflects a transitory emotional state with the scale consisting of 20 statements that ask the subject to describe rate feelings at a particular moment on a four-point scale ranging from 'not at all' to 'very much so'. In contrast, trait anxiety reflects relatively stable individual differences in anxiety with the scale consisting of 20 statements describing how the subject generally feels rated on a four-point scale ranging from 'almost never' to 'almost always'. Scores on the STAI have a direct interpretation: high scores on their respective scales mean more trait or state anxiety and low scores mean less. The STAI has been validated in older populations with adequate internal consistency (α = 0.88 to 0.94) and test-retest reliability (r = 0.51 to 0.58) [38].

#### Pain Catastrophizing Scale (PCS)

This scale measures three dimensions of pain catastrophizing (rumination, magnification, and helplessness). It is a 13-item self-report scale that assesses the degree to which subjects have different thoughts and feelings when experiencing pain and is determined with a five-point frequency scale ranging from 'not at all' to 'all the time'. Higher scores indicate more pain catastrophizing. The PCS was originally developed by Sullivan and colleagues [39] and has adequate reliability in adult samples (α = 0.93 to 0.95; test-retest r = 0.75) with good convergent validity with self-reported anxiety (r = 0.32) [40,41].

#### Social Provisions Scale (SPS)

This scale measures the construct of social support [42,43] and has been validated for usage with populations of older adults with convergent validity (r = 0.18 to 0.22) to morale and friend contact [44]. The SPS has 24 items that are rated as 1 (strongly agree) to 4 (strongly disagree) with half of the items worded as positive and half as negative. Negative items are reversed for scoring to allow for higher scores to indicate more social support.

### Physical activity

#### Accelerometer

An ActivPal accelerometer (PAL Technologies Ltd, 50 Richmond Street, Glasgow G1 1XP, Scotland, UK) was used to objectively record physical activity. Subjects wore the accelerometer for 1 week or until their surgery date, whichever came first. Subjects with less than 2 days of measurement (due to surgery date or device malfunction) were excluded from this analysis resulting in a subsample size of 176 subjects. The range of measurement was 2 to 11 days (mean = 5.91 ± 1.77). The subjects wore the accelerometer taped to the anterior thigh as directed by the manufacturer and were instructed to wear the device continuously, removing the device only for water activities (bathing/swimming) as the device was not waterproof. The ActivPal uses proprietary algorithms to calculate the amount of time a subject spends sitting, standing, and walking and also provides an estimate for energy expenditure (METS). Accelerometers have been used in populations of older adults with OA [18,45]. Accelerometers have adequate validity in older adults (r = 0.6) when compared to a criterion measure of energy expenditure [46] and high reliability between units (ICC = 0.97 to 0.99) for the raw data of activity counts and steps [47]. While new research suggests an overall ICC of 0.57 for the ActivPal METS calculation compared to indirect calorimetry [48] the METS equation was more accurate at slower walking speeds and while sedentary which are likely in our population of older adults with arthritis.

### Statistical analysis

Univariate and multivariate analyses were conducted using SPSS for Windows V. 17.0 (SPSS, Chicago, IL, USA). Univariate t tests were first used to determine if there were significant differences between women and men for the following variables: age, BMI, medication intake, pain, perceived function, psychosocial variables, functional tests, and quantitative sensory measures. A χ^2 ^test was used for the categorical variables such as race, marital status, education, income, pain duration, OA grade, contralateral pain or OA, and depression. Bonferroni adjustments were made for multiple univariate comparisons on the same measure (SF-36, KOOS, accelerometry) to control for error. Multiple linear regression was conducted using a stepwise selection procedure to determine the best combination of variables to explain the variation in pain during walking, distance walked during the gait speed test, and average METS/day calculated by accelerometry for the population as a whole and separately for men and women.

## Results

Demographic data for both women and men are shown in Table 1. Women had higher BMI's on average than men with indexes of 35.43 ± 7.59 and 33.19 ± 6.59 for women and men, respectively (*P *= 0.04). Women also had significantly lower OA grades on the Kellgren-Lawrence Scale when compared to men (*P *= 0.03) No significant differences (*P *> 0.05) between women and men were observed for age, race, marital status, education, income, duration of knee pain, contralateral knee pain or OA, and intake of opiate and non-opiate pain medications. It should be noted, however that income (*P *= 0.05), pain at rest (*P *= 0.08) and non-opiate medication usage (*P *= 0.06) were close to statistical significance.

**Table 1:** Demographics

		Women	Men	*P *value
Sample (n = 208)		66.3% (n = 138)	33.7% (n = 70)	
Age		61.92 (10.03)	61.66 (9.92)	0.86
Race	White	93.48%	92.86%	0.23
	Black	4.35%	2.86%	
	Other	2.17%	1.43%	
Marital status	Married	55.80%	60%	0.54
	Single	35.51%	31.43%	
Education	High school	30.43%	28.57%	0.80
	College	60.14%	61.43%	
Income	US$0 to US$19,999	28.26%	14.29%	0.05
	US$20,000 to US$39,999	17.39%	21.43%	
	US$40,000 to US$59,999	13.77%	11.43%	
	US$60,000+	23.19%	37.14%	
Duration of knee pain (months)	0 to 35 months	23.19%	25.71%	0.86
	36 to 59 months	19.57%	21.43%	
	> 60 months	56.52%	52.86%	
OA grade	3	32.61%	18.57%	0.03*
	4	60.14%	74.29%	
Contralateral knee pain		78.26%	72.86%	0.39
Contralateral knee OA		14.49%	15.71%	0.84
BMI		35.43 (7.59)	33.19 (6.59)	0.04*
Pain at rest (0 to 20)		3.77 (4.36)	2.67 (3.81)	0.08
Non-opiate medication (acetaminophen equivalent)		980.84 (1,107.9)	681.79 (1,048.53)	0.06
Opiate medication (morphine equivalent)		6.76 (15.71)	3.86 (12.19)	0.14

BMI = body mass index; OA = osteoarthritis.

* = significance < 0.05

### Pain

**Figure 1:**
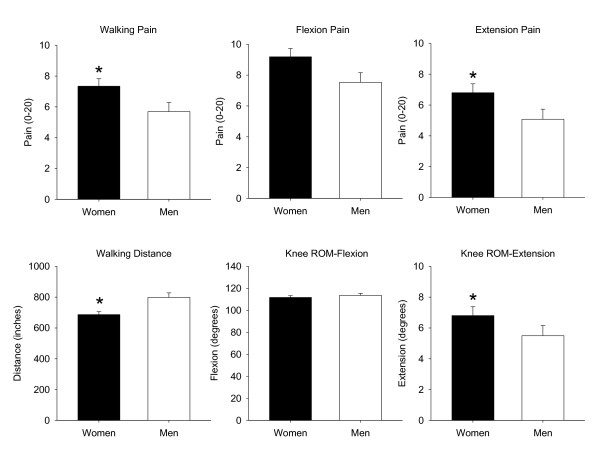
**Pain and function scores between men and women**. Significant differences occurred for walking pain, extension pain, walking distance and knee extension (*P < 0.05).

Pain at rest (0 to 20 NRS) did not vary significantly between women and men with the average resting pain measured as 3.77 ± 4.37 and 2.67 ± 3.81, respectively (*P *= 0.08). Pain intensity during function tasks (gait speed test, active flexion and extension) is shown in Figure 1. Women had significantly higher pain intensity (7.34 ± 5.69) than men (5.69 ± 4.95) (*P *= 0.04) during the gait speed test. Women also reported higher pain intensity during active knee extension: women 8.40 ± 6.43 and men 5.93 ± 5.44 (*P *= 0.004) but not during active flexion (*P *= 0.06). The BPI Intensity scores showed women had significantly more intense pain averaging 5.4 ± 1.69 compared to men's average of 4.41 ± 2.02 (*P *= 0.001). Women also had significantly worse pain than men on both the SF-36 Pain subscale and KOOS Pain subscale (note that a lower score for women indicates more impairment or worse pain) (*P *< 0.05) (see Table 2).

**Table 2:** Self-reported pain and function for Short-Form 36 (SF-36) and Knee Injury and Osteoarthritis Outcome Score (KOOS); higher scores indicate better health/fewer impairments

Results	Women, average (SD)	Men, average (SD)	*P *value
BPI Severity	5.40 (1.69)	4.41 (2.02)	0.001*
SF-36 Pain	34.68 (18.23)	45.98 (23.57)	< 0.001*
SF-36 PF	31.08 (19.48)	38.87 (24.02)	0.018*
KOOS Pain	44.08 (18.71)	50.77 (17.58)	0.020*
KOOS ADL	52.51 (19.47)	61.09 (22.05)	0.007*

ADL = activity in daily living; BPI = Brief Pain Inventory; PF = physical function.

* = significance < 0.05

Significant predictors of pain with walking utilizing regression included pain at rest, degrees of active knee extension, state anxiety, and pressure pain threshold. These variables explain 24.1% of the variance in pain with movement (Table 3). Variables not significant in the model included sex, age, BMI, OA grade, depression, medication usage, pain duration, pain catastrophizing, active knee flexion, and the thermal threshold and tolerance. These predictor variables were then analyzed for women and men as separate groups and it was found that pain at rest, active knee extension, and pressure pain thresholds were predictive of pain with movement for women (R^2 ^= 0.268) while pain at rest and pressure pain thresholds were predictive for men (R^2 ^= 0.181); thus, active knee extension was an additional predictor for women. Variables not significant in the full model included sex, age, BMI, OA grade, depression, medication usage, pain duration, pain catastrophizing, active knee flexion, and the thermal threshold and tolerance.

**Table 3:** Pain regression (dependent variable was pain with gait speed 0 to 20 NRS)

Predictor variable	β	Standard error	*P *value
All subjects:			
Pain at rest	0.400	0.096	< 0.001
Knee extension	0.193	0.057	0.001
State anxiety	0.075	0.042	0.081
PPT	-0.005	0.002	0.024
	F value	Significance	Model fit
Overall model	12.713	< 0.001	R^2 ^= 0.241
Women:			
Pain at rest	0.421	0.103	< 0.001
Knee extension	0.243	0.062	< 0.001
PPT	-0.010	0.004	0.018
Women only model	15.295	< 0.001	R^2 ^= 0.267
Men:			
Pain at rest	0.422	0.151	0.007
PPT	-0.005	0.003	0.086
Men only model	6.408	0.003	R^2 ^= 0.181

NRS = numerical rating scale; PPT = pressure pain threshold.

### Function

Functional measures are presented in Figure 1 and Table 2. Women had significantly poorer active knee extension then men with a loss of 6.83° from full extension versus 5.07° for men (*P *= 0.048) but not differ significantly from men on active knee flexion (*P *= 0.43). The distance traveled during the gait speed test differed significantly between women and men with women averaging a distance traveled of 111.7 inches less than men (*P *= 0.001). Self-report function measures using the SF-36 physical function (PF) subscale showed women have significantly worse perceived function than men (lower scores) with scores of 31.08 ± 19.48 and 38.87 ± 24.02, respectively (*P *= 0.018). Similarly, women have significantly worse perceived function on the KOOS ADL subscale with scores of 52.51 ± 19.47 for women and 61.09 ± 22.05 for men (*P *= 0.007).

Significant predictors of function, measured by distance on the gait speed test, utilizing regression included knee flexion and extension, age, sex, current opioid medication usage, pain duration, pain catastrophizing, BMI, and HPThr. These variables explained 34.5% of the variation in function (Table 4). These predictor variables when analyzed separately for women and men showed age, active knee flexion, opioid medications, pain catastrophizing, and BMI were predictive of gait speed for women (R^2 ^= 0.312) and active knee extension and age (R^2 ^= 0.129) were predictive in men. Variables not significant in the model included OA grade, depression, non-opioid medications, anxiety, resting pain, pressure pain thresholds, and heat pain tolerance.

**Table 4:** Function regression (dependent variable was gait speed test)

Predictor variable	β	Standard error	*P *value
All subjects:			
Knee extension	-5.013	2.555	0.051
Age	-8.306	1.695	< 0.001
Knee flexion	3.374	1.180	0.005
Sex	-80.966	34.717	0.021
Opioid medications	-2.489	1.083	0.023
Pain duration	38.014	18.334	0.040
Pain catastrophizing	-3.294	1.644	0.047
BMI	-4.283	2.393	0.075
Heat pain threshold	10.090	5.278	0.058
	F value	Significance	Model fit
Overall model	9.062	< 0.001	R^2 ^= 0.345
Women:			
Age	-9.749	1.802	< 0.001
Knee flexion	3.170	1.094	0.004
Opioid medications	-2.829	1.097	0.011
Pain catastrophizing	-3.538	1.847	0.058
BMI	-4.525	2.625	0.087
Women only model	10.630	< 0.001	R^2 ^= 0.312
Men:			
Knee extension	-13.302	5.159	0.012
Age	-4.755	2.830	0.098
Men only model	4.358	0.017	R^2 ^= 0.129

BMI = body mass index.

### Pain sensitivity (quantitative sensory tests)

PPT, HPThr, and HPTol data are presented in Table 5. Women had significantly lower pain thresholds (greater sensitivity) to pressure and heat stimuli and lower pain tolerance to heat stimuli than men. On the affected knee, pressure pain thresholds were 234.35 ± 112.73 kPa for women and 373.23 ± 207.03 kPa for men (*P *< 0.001), heat pain thresholds were 42.86 ± 3.21°C for women and 44.71 ± 2.78°C for men (*P *< 0.001), and heat pain tolerance were 46.70 ± 2.70°C for women and 48.51 ± 1.59°C for men (*P *< 0.001). Utilizing simple regression with only gender in the model resulted in gender explaining 16% of the variability in PPT (β = -138.87, *P *< 0.001), 7.6% of HPThr (β = -1.86, *P *< 0.001), and 11.5% of HPTol (β = -1.81, *P *< 0.001).

**Table 5:** Quantitative sensory testing

Sensory test	Women, average (SD)	Men, average (SD)	*P *value
Algometer (kPa), affected knee	234.35 (112.73)	373.23 (207.03)	< 0.001*
Algometer (kPa), contralateral knee	255.74 (117.75)	414.40 (209.31)	< 0.001*
HPThr (°C), affected knee	42.86 (3.21)	44.71 (2.78)	< 0.001*
HPThr (°C), contralateral knee	42.73 (3.03)	44.70 (2.30)	< 0.001*
HPTol (°C), affected knee	46.70 (2.70)	48.51 (1.59)	< 0.001*
HPTol (°C), contralateral knee	47.01 (2.04)	48.70 (1.53)	< 0.001*

HPThr = heat pain threshold; HPTol = heat pain tolerance.

* = significance < 0.05

### Psychosocial variables

Women and men did not significantly differ on any of the psychosocial variables measured in this study (Table 6). Depression rates were the same for women and men (15.3% and 15.9%, respectively). Trait anxiety showed women scored an average of 34.88 ± 10.15 while men scored an average of 32.81 ± 9.85 (*P *= 0.18), which was similar to the scores of state anxiety of 34.71 ± 8.94 for women and 32.61 ± 10.02 for men (*P *= 0.15). PCS scores were also similar for women and men with scores of 11.81 ± 9.52 and 11.06 ± 11.41, respectively (*P *= 0.63). Women and men also reported a similar degree of social support as measured by the SPS with average scores of 80.12 ± 10.95 for women and 80.92 ± 9.89 for men (*P *= 0.63).

**Table 6:** Psychosocial measures

Psychosocial measure	Women, average (SD)	Men, average (SD)	*P *value
Depression	15.3%	15.9%	0.91
Trait anxiety	34.88 (10.15)	32.81 (9.85)	0.18
State anxiety	34.71 (8.94)	32.61 (10.02)	0.15
Catastrophizing	11.81 (9.52)	11.06 (11.41)	0.63
Social provisions	80.12 (10.95)	80.92 (9.89)	0.63

### Accelerometry

The accelerometer results are presented in Table 7. There were no significant differences between women and men for the average METS/day (32.33 ± 1.24 vs 32.51 ± 1.25; *P *= 0.35), average transitions/day (51.08 ± 16.82 vs 50.53 ± 15.80; *P *= 0.84), average steps/day (4,544.36 ± 2,725.11 vs 5,086.10 ± 2,905.46; *P *= 0.23), or average time spent vertical/day (3.50 ± 1.80 vs 3.32 ± 1.70 h; *P *= 0.52).

**Table 7:** Accelerometer descriptive results

Accelerometer measures	Women, average (SD)	Men, average (SD)	*P *value
Average METS/day	32.33 (1.24)	32.51 (1.25)	0.35
Average transitions/day	51.08 (16.82)	50.53 (15.80)	0.84
Average steps/day	4,544.36 (2,725.11)	5,086.10 (2,905.46)	0.23
Average time vertical/day (h)	3.50 (1.80)	3.32 (1.70)	0.52

METS = metabolic equivalent tasks.

Variables that significantly explained the variation in average METS/day included BMI, age, OA grade, depression, SF-36 PF, and KOOS Pain. These variables explained 35.6% of the variation in METS/day for all subjects, regardless of gender (Table 8). Gender was not significant in explaining the average METS/day. Other variables that were not significant in the model were: pain intensity at rest and during function measures, gait speed distance, degrees of active flexion and extension, pain catastrophizing, state and trait anxiety, social support, analgesic intake, and pain duration. Results (data not shown) were similar when using average steps/day as the outcome variable.

**Table 8:** Accelerometer regression (dependent variable was average metabolic equivalent tasks (METS)/day)

Predictor variable	β	Standard error	*P *value
All subjects:			
BMI	-0.074	0.013	< 0.001
Age	-0.039	0.010	< 0.001
OA grade	-0.512	0.171	0.003
Depression	-0.618	0.301	0.042
SF-36 PF	0.011	0.005	0.019
KOOS Pain	-0.011	0.006	0.073
	F value	Significance	Model fit
Overall model	12.086	< 0.001	R^2 ^= 0.356
Women:			
SF-36 PF	0.016	0.006	0.004
BMI	-0.074	0.015	< 0.001
Age	-0.050	0.011	< 0.001
OA grade	-0.466	0.187	0.019
Women only model	13.284	< 0.001	R^2 ^= 0.354
Men:			
BMI	-0.079	0.024	0.002
Age	-0.033	0.017	0.061
Men only model	6.269	0.004	R^2 ^= 0.218

BMI = body mass index; KOOS = Knee Injury and Osteoarthritis Outcome Score; OA = osteoarthritis; PF = physical function; SF-36 = Short-Form 36.

## Discussion

The results of this study show for the first time a distinct gender difference for pain during movement but not for pain at rest. We also show for the first time that psychosocial variables (depression, anxiety, pain catastrophizing, and social provisions) are similar between men and women with late-stage osteoarthritis. Similar to prior studies, pain sensitivity, perceived function and function tests are reduced in women compared to men (see [14]). Surprisingly, while women had significantly worse pain and more impaired function than men, their actual physical activity levels (accelerometry) did not significantly differ and their OA grade was significantly lower, that is, less severe. This study developed predictive models to explain physical activity, function, and pain in people with OA using a comprehensive biopsychosocial approach. When both men and women were considered, physical activity levels were predicted by BMI, age, OA grade, depression, SF-36 PF, and KOOS Pain; pain during movement was predicted by pain at rest, knee extension, state anxiety and pressure pain thresholds; Function was knee flexion and extension, age, sex, opioid medication usage, pain duration, BMI, and heat pain threshold. Different predictive factors were found when the analysis was run with the men and women separately. We therefore, for the first time, were able to model physical activity levels, pain and function with multiple biopsychosocial variables, and to determine if there were differences between men and women in these variables.

Prior studies have modeled a number of different outcomes in people with OA to determine relevant factors that can predict outcomes [49-54]. Of direct relevance, in a sample of 168 OA subjects, sex predicted pain related outcomes (pain, disability and pain behaviors) and catastrophizing mediated the relationship between sex and OA pain-related outcomes [55]. Further, in a study with 106 OA subjects, pain catastrophizing was a significant predictor of pain severity, disability, and function measured by gait [53]. We extended these studies and showed for the first time that quantitative sensory testing (PPTs) predicted pain with movement, both evoked pain measures. We also show that for pain with movement that knee range of motion was an additional predictor for women but not for men. We also extend these findings and show that for function women had more predictors than men, which included opioid medications and pain catastrophizing as predictors of function only for women. However, our studies do not completely agree with prior studies in that pain catastrophizing did not predict pain during movement or physical activity. Differences in sample size (106 vs 268), OA severity (early vs Pre-total knee arthroplasty), and outcomes measures for pain (AIMS and observed pain behaviors vs pain with movement) and function (self-report vs gait speed or accelerometry) could underlie the lack of agreement between prior and the current study.

### Pain during rest and movement

The current study found no significant gender difference in resting pain but significant gender differences for pain during movement and self-reported pain as measured by surveys (BPI, SF-36 Pain subscale, and KOOS Pain subscale). This is consistent with larger studies that show worse pain in women compared to men using the Knee Society Score survey instrument and the AIMS [5,55]. Perceived pain measured by surveys reflects both pain at rest and pain during function. The results of this study suggest that pain during function has the largest impact on the sex differences found when measuring pain using self-report survey instruments.

The current study also found that women had lower Kellgren-Lawrence grades when compared to men, despite higher pain. These results are in agreement with prior studies that show women have more severe symptoms at the same Kellgren-Lawrence grades when compared to men [56]. This difference in pain in relation to OA grade is not manifested in early knee OA [18]. It has been hypothesized that women may have more severe osteoarthritis than men at the presurgical stage and wait longer to have surgery [8,57,58]. In fact, women lose articular cartilage from the proximal tibia at four times the annual rate of men and from the patella at a threefold greater rate [59]. In contrast, the current study showed women have less severe Kellgren-Lawrence scores with a similar duration of pain just prior to surgery. These data suggest that women have higher pain despite lower radiographic evidence of OA and wait a similar length of time to have surgery.

### Functional differences in OA

In the current study, women had more deficits on self-reported function on the SF-36 PF subscale and KOOS ADL subscale when compared to men, which is in agreement with prior literature [60]. Similar differences have also been reported on the Knee Society and AIMS [3,5,6,55,58]. The current study showed reduced ability to perform the gait speed test and reduced knee active range of motion. These data are in agreement and extend prior studies that show reduced function on the 6-minute walk test, the timed up and go test, and stair climbing test times [7,8]. The functional differences in knee OA subjects may in part be due to known differences in quadriceps muscle strength between women and men [8,9].

The current study showed that physical activity levels measured by accelerometry were similar between women and men immediately prior to surgery, despite differences in perceived function and functional tasks. This is in contrast to prior studies that show lower physical activity levels in women with early OA compared to men [18]. The gender differences in physical activity levels are present in healthy populations, where men spend more time in activities of higher intensity than women [61,62]. Just prior to surgery, these differences in physical activity levels seem to disappear. However, men with OA have better performance on timed walk tests and stairs than women [63], which agrees with the results of the current study. Physical function tests are also similar to perceived function in patients with OA. This would suggest that physical function tests and perceived function are similar constructs, but that physical activity, measured by accelerometry, is a different construct. These results also suggest that women with late stage knee OA continue to move as much as men despite more pain during movement, greater pain sensitivity, and less functional ability.

### Gender differences in pain sensitivity

The current study, in concurrence with prior literature, shows clear gender differences in pain sensitivity with women having greater sensitivity to heat, cold, and mechanical pressure [10-12]. Across the lifespan, women are more sensitive to heat pain with a nociceptive threshold 1.6°C lower in women than in men [13]. These differences between women and men also occur for pressure pain thresholds; however, the differences tend to converge with age, with no gender difference in pressure pain thresholds at 50 to 70 years [13]. We found the differences in pressure pain thresholds maintained in our population suggesting greater mechanical pain sensitivity of the deep tissue in women when compared to men when a chronic pain condition such as OA is present. This relationship of greater clinical and experimental pain in women has recently been shown in a chronic shoulder pain population [64].

### Psychosocial variables

The current study showed similar scores between women and men for depression, state anxiety, trait anxiety, pain catastrophizing, and perceived social support. It is often noted that women have a higher prevalence of depression [65]. However, we noted no significant difference with around 15% of both women and men screening positive for depression. This prevalence rate is similar to prior studies in chronic pain populations [14,66]. However, one study found that depression tendency in older Chinese patients with OA explained a portion of the gender differences in pain [67]. The current study similarly, shows that depression explains a portion of physical activity levels in people with late-stage OA. Thus, depression may be related to not only pain, but also function in people with OA.

Sex differences in anxiety are controversial with some studies finding significant differences while others do not [68-70]. Some report that men with higher anxiety also have higher pain intensity [68] while other research suggests that this relationship is actually stronger in women [70]. People with OA have higher anxiety than the general population, which is associated with higher pain intensity, worse symptoms, and greater healthcare utilization [71]. However, based on the current study, these higher anxiety rates appear to occur similarly among women and men.

Pain catastrophizing has also shown a mixed relationship in pain research with some studies showing no gender differences [15,72,73] while others showing women have significantly higher pain catastrophizing [55]. Prior work shows that pain catastrophizing may increase daily pain recall, but does not explain differences in experimental pain [72]. The differences between studies could be due to studying different populations (younger vs older; experimental vs clinical pain) or using different measurement tools for catastrophizing. In people with OA, higher pain catastrophizing scores are associated with greater pain and disability [49,55], pain 6 weeks after total knee replacement [50], and poor outcome 6 months after total knee replacement [74]. The current study shows similar catastrophizing scores between women and men, and pain catastrophizing did not explain differences in physical activity levels. Thus, while pain catastrophizing is clearly a valuable construct that explains pain in people with OA, there was no sex differences observed in this population.

### Limitations

Our subjects were recruited from a large teaching hospital, which may include a different patient population than other clinical settings. There are many ways to analyze the differences between women and men. For our regressions explaining pain, function, and accelerometry, we did not perform further analyses to see if the predictor variables were different for women and men. That will be focused on and analyzed further as we follow this population through the preoperative to the postoperative period. The usage of accelerometry is beneficial to help in understanding actual levels of physical activity, but there are limitations in the validity of the METS equation as a subject performs higher levels of physical activity. Further research will focus on how the ActivPAL variables differ between our OA population and healthy controls.

## Conclusions

Our results clearly show that women have greater pain, greater pain sensitivity, and reduced function when compared to men. The role of sex needs to be further examined to determine if these pain differences are due to hormonal differences, socialization, or other factors. Further, we show similar scores between women and men on psychosocial variables including depression, anxiety, pain catastrophizing, and social support suggesting that observed gender differences in pain and function are not related to psychosocial differences.

### Clinical significance

Clinically, these data suggest that treatments should place a greater emphasis on pain management and improving function, particularly in women with OA. Our data show no sex differences in medication usage, despite higher pain in women. This could suggest that women are less sensitive to current pain medication strategies, and/or that alternative pharmacological and non-pharmacological pain management strategies would be more effective in women. Sex differences in predictors of pain and function further suggest pain management strategies should be individualized based on patient characteristics that include sex.

## Competing interests

The authors declare that they have no competing interests.

## Authors' contributions

KS designed the study, analyzed the data and helped draft the manuscript. BR designed the study and reviewed the manuscript. ST performed the experiment, analyzed the data, performed statistical analysis and helped draft the manuscript. NC performed the experiment and assisted with data analysis. WA assisted with data collection, data analysis, and literature review. All authors read and approved the final manuscript.
